# Neural Basis of Anxiety in Dementia With Lewy Bodies

**DOI:** 10.1002/gps.70150

**Published:** 2025-09-03

**Authors:** Naohiro Kimura, Yoshihiro Chadani, Ryo Kawai, Ryoko Fujito, Hideki Kanemoto, Ryuichi Takahashi, Tetsuo Kashibayashi, Shunichiro Shinagawa, Kenji Tagai, Kazunari Ishii, Manabu Ikeda, Hiroaki Kazui

**Affiliations:** ^1^ Department of Neuropsychiatry Kochi Medical School Kochi University Nankoku Japan; ^2^ Department of Rehabilitation Atago Hospital Branch Kochi Japan; ^3^ Department of Psychiatry The University of Osaka Graduate School of Medicine Suita Japan; ^4^ Health and Counseling Center The University of Osaka Toyonaka Japan; ^5^ Dementia‐related Disease Medical Center Hyogo Prefectural Rehabilitation Hospital at Nishi‐Harima Tatsuno Japan; ^6^ Department of Psychiatry Jikei University School of Medicine Tokyo Japan; ^7^ Department of Radiology Kindai University Faculty of Medicine Osakasayama Japan

**Keywords:** anxiety, behavioral and psychological symptoms of dementia, dementia with Lewy bodies, fluctuating cognition, right supramarginal gyrus, statistical parametric mapping, supportive clinical features

## Abstract

**Objective:**

The association between core clinical features and anxiety and the neural basis of anxiety in patients with dementia with Lewy bodies (DLB) are unknown. Therefore, this study examined the core clinical features associated with anxiety in DLB and identified the brain regions associated with anxiety using statistical imaging analysis.

**Methods:**

This study was conducted using a part of the data from “The Japan multicenter study: Behavioral and psychological symptoms Integrated Research in Dementia‐Retrospective Neuroimaging part”. Overall, 40 patients with probable DLB whose clinical dementia rating score was either 0.5 or 1 were included in this study. Anxiety was evaluated using the Neuropsychiatric Inventory (NPI). The incidence of each of the 4 core features was compared between patients with and without anxiety, and the brain regions associated with anxiety were examined using single‐photon emission computed tomography data.

**Results:**

Patients with DLB with anxiety had a significantly higher percentage of fluctuating cognition than those without anxiety. The NPI anxiety score was significantly negatively correlated with regional cerebral blood flow in the right supramarginal gyrus in patients with DLB.

**Conclusion:**

Anxiety in DLB is associated with fluctuating cognition. It is also likely that the brain regions associated with anxiety in DLB are potentially influenced by the neurofunctional characteristics of DLB, in which the parietal lobes are more likely to be impaired.

## Key Points

- Patients with dementia with Lewy bodies (DLB) who have anxiety have significantly higher rates of fluctuating cognition than those without anxiety.
- Anxiety in patients with DLB is significantly associated with reduced regional cerebral blood flow (rCBF) in the right supramarginal gyrus.
- This is the first study to identify the neural basis of anxiety in patients with DLB using rCBF single‐photon emission computed tomography imaging data and to demonstrate its association with fluctuating cognition.

## 1 Introduction

Dementia with Lewy bodies (DLB) is the second most common form of neurodegenerative dementia following Alzheimer's disease (AD) [1]. The neuropathology of DLB is characterized by the accumulation of Lewy bodies in the brain [2], and presents core clinical features of fluctuating cognition with pronounced variations in attention and alertness, recurrent visual hallucinations that are typically well formed and detailed, rapid eye movement (REM) sleep behavior disorder (RBD), and idiopathic parkinsonism [3]. Moreover, in the early stage, patients with DLB may develop various and severe behavioral and psychological symptoms of dementia (BPSD) [4]. Anxiety, delusions, depression, and apathy are among the BPSDs included as supportive clinical features in the current consensus clinical diagnostic criteria for DLB [3].

Among the 4 supportive clinical features of DLB, delusions and apathy are more frequently observed than anxiety and depression; however, similar to depression, anxiety is common, accounting for 33.9%, 36.7%, and 48.4% of patients with mild cognitive impairment, mild dementia, and moderate dementia, respectively [4]. Moreover, anxiety and depression has been reported to appear as early as 5 years before diagnosis in > 30% of patients with prodromal DLB [5]. Furthermore, anxiety in DLB is more strongly associated with caregiver burden than delusions, depression, or apathy [6]. Regarding the pharmacological treatment of anxiety in DLB, only two studies, that is, one with rivastigmine [7] and the other with yokukansan [8], have demonstrated effectiveness. However, anxiety may persist in patients with DLB even after the administration of medication [8]. Antipsychotics have been proven effective for treating anxiety in patients with AD [9, 10] and vascular dementia [10]; however, these medications must be administered with extreme caution in patients with DLB owing to their high susceptibility to adverse effects [3]. Therefore, establishing effective treatment strategies for anxiety in DLB is urgently needed.

Associations between BPSDs in DLB and regional cerebral blood flow (rCBF) SPECT imaging findings have been reported. Delusions in DLB have been linked to relatively increased rCBF in the right rostral medial frontal cortex, bilateral middle frontal gyrus, right inferior frontal gyrus, left medial superior frontal gyrus, and left middle frontopolar gyrus [11]. Depression has been linked to increased rCBF in the left angular gyrus and right upper precuneus [12]. Furthermore, visual hallucinations in DLB are strongly associated with reduced occipital lobe rCBF [11, 12], and treatment with donepezil has been reported to improve occipital lobe rCBF, accompanied by a reduction in visual hallucinations [13]. These findings suggest that rCBF SPECT facilitates understanding of the neural substrates of BPSDs in DLB and is valuable in evaluating treatment responses. However, the neural basis of anxiety in DLB remains poorly understood.

Understanding the association between core clinical features and BPSDs may facilitate in developing appropriate intervention strategies in patients with DLB. Regarding anxiety, a previous study reported that experimentally inducing an anxious mood triggered pareidolic illusions, which are associated with visual hallucinations, in patients with DLB [14]. However, the study did not examine the associations between anxiety and core clinical features other than visual hallucinations. Another study examined the association between anxiety and the 3 core clinical features except for RBD, and showed no significant associations between anxiety and the 3 core clinical features [15]. Therefore, clarifying the association between anxiety and all core clinical features in DLB remains a crucial issue.

Based on these backgrounds, the present study aimed to identify brain regions associated with anxiety in DLB using statistical imaging analysis and to examine which core clinical features are related to anxiety.

## 2 Methods

### 2.1 Procedures and Participants

This study was part of a large multicenter project conducted in Japan, “The Japan multicenter study: Behavioral and psychological symptoms Integrated Research in Dementia‐Retrospective Neuroimaging part (J‐BIRD‐RN).” This retrospective observational study was conducted without intervention and in compliance with national legislation and the Declaration of Helsinki. All patient information was anonymized and stored as unlinked data before analysis to prevent the disclosure of personal information. The ethics committee of Kochi University Hospital and Jikei University Hospital approved this study. At the outpatient departments of the J‐BIRD‐RN centers, patients were examined by psychiatrists and neurologists specializing in dementia, as well as routine laboratory tests and standard neuropsychological examinations. Moreover, they were evaluated in terms of activities of daily living. Based on these data, clinical dementia rating (CDR) scores were calculated to determine dementia severity [16]. BPSDs were evaluated using the Neuropsychiatric Inventory (NPI) [17]. Additionally, brain magnetic resonance imaging (MRI) and/or cerebral perfusion studies using single‐photon emission computed tomography (SPECT) were performed. Probable DLB was diagnosed according to the International Diagnostic Criteria of the 2005 edition [18] until July 2017 and the 2017 edition [3] after August 2017.

This study used part of the data enrolled at the Memory Clinic of the Department of Neuropsychiatry in two centers, including Osaka University and Kochi University Medical Hospitals. Specifically, 44 consecutive cases of DLB who visited either hospital between April 2015 and December 2021, had MRI and iodoamphetamine (IMP) SPECT, and whose CDR score was either 0.5 or 1 were included in this study. Further, of the 44 patients, 4 were excluded (2 who had no Alzheimer's Disease Assessment Scale‐Cognitive component (ADAS) data and 2 who were ambidextrous). Therefore, data of the remaining 40 patients (20 males, 20 females, all right‐handed) were used for the present analyses. Of the 40 patients, dopamine transporter SPECT was performed in 36 patients, with 18 patients demonstrating abnormal findings. ^123^I‐metaiodobenzylguanidine myocardial scintigraphy was performed in 37 patients, with 20 patients exhibiting abnormal findings. Both tests revealed abnormal findings in 9 patients, while both tests revealed normal findings in 9 patients. Neither test was performed in 2 patients.

### 2.2 Clinical Assessments

#### 2.2.1 Assessments of Anxiety

We here used the composite score of the anxiety item of the NPI as the anxiety score [17]. The NPI encompasses the following 10 items: delusions, hallucinations, agitation, depression, anxiety, euphoria, apathy, disinhibition, irritability, and aberrant motor behavior. Caregivers of patients were asked to rate the frequency and severity of each symptom. The frequency of each item was classified into grades 0–4, and the severity of each item was classified into grades 0–3, with 0 indicating absence of symptom and higher scores indicating higher frequency or worse symptom. A composite score was obtained for each symptom by multiplying frequency and severity scores, with a maximum score of 12 for each item. A higher NPI score indicated higher Neuropsychiatric Symptoms severity.

In this study, patients with DLB with NPI anxiety scores of at least one point were classified into Anxiety+ group and those with NPI anxiety scores of 0 point were classified into Anxiety− group.

#### 2.2.2 Assessments of Clinical Features

Data documenting clinical findings for the presence or absence of the 4 core clinical features in DLB (fluctuating cognition, visual hallucinations, RBD, and parkinsonism), as assessed by dementia specialists, were used.

For neuropsychological function assessment, data of Mini‐Mental State Examination (MMSE) [19] and the Japanese version of ADAS [20] scores were used. Higher scores indicate better performance in MMSE and worse performance in ADAS. Data of the Serial sevens test included in the MMSE were used as measures of attentional function. Performance in the double pentagon copying task in the MMSE was used as a measure of visuoconstructional ability and was categorized as either pass or fail depending on whether the figure was correctly drawn. Each of the 11 ADAS items was categorized into the following 4 domains: memory, orientation, language, and act [21]. In the 4 areas, the evaluation points for memory loss had a total of 27 points as follows: word reproduction function decrease (10 points), word recognition function decrease (12 points), and the regeneration of test instruction decrease (5 points). The evaluation points for language function decline had a total of 20 points as follows: speech (language) ability decrease (5 points), auditory understanding decrease (5 points), word recall (5 points), and finger and article name function decrease (5 points). The evaluation points for the action function decrease had a total of 15 points as follows: verbal command function decrease (5 points), constructional praxis function decrease (5 points), ideational praxis function decrease (5 points), and disorientation function decrease was 8 points only of the orientation function decrease (8 points).

### 2.3 Neuroimaging Assessments

#### 2.3.1 SPECT Data Acquisition

Brain perfusion images were obtained using N‐isopropyl‐p‐[^123^I] IMP and SPECT in the J‐BIRD‐RN–nominated institutions (Siemens Symbia T2 SPECT/CT at Kochi University Hospital and Siemens Healthcare Symbia T‐6 and Siemens Healthcare Symbia Intevo at Osaka University Hospital). The imaging scan was started in the resting state, 15 min after the injection of 111–222 MBq (3–6 mCi) of ^123^I‐IMP (Supporting Information S1).

#### 2.3.2 MRI Data Acquisition

In this study, three‐dimensional T1‐weighted images were obtained at J‐BIRD‐RN–nominated institutions (Philips Ingenia 3.0 T and GE SIGNA Architect 3.0 T at Kochi University Hospital and SIGNA EXCITE HD 1.5 T and SIGNA Explorer 1.5 T at Osaka University Hospital).

#### 2.3.3 Neuroimaging Data Analysis

In this study, anatomical normalization and statistical processing of the SPECT images were performed using Statistical Parametric Mapping 12 (SPM 12; Wellcome Department of Cognitive Neurology, London, United Kingdom).

To normalize MR images, the diffeomorphic anatomical registration through exponentiated lie algebra (DARTEL) algorithm [22] implemented in the SPM 12 was used. Briefly, MR images were segmented into the gray matter, white matter, and cerebrospinal fluid space on the basis of the tissue probability maps. A DARTEL template was constructed using the segmented gray matter images of all participants. Subsequently, each original MR image and co‐registered SPECT image were transformed into a stereotactic anatomical space with deformation parameters and template that were constructed in the DARTEL deformation process of the MR images. Lastly, each SPECT image was normalized with cerebellar counts. After smoothing images with 8 mm Gaussian filter, SPM 12 was used for the correlation analysis between the NPI anxiety score and rCBF in DLB considering age, sex, CDR, and SPECT model as covariates. If there were symptoms within the 4 core features with percentages significantly differing between Anxiety+ and Anxiety− groups, the presence or absence of those symptoms would also be included in the covariate. To improve the sensitivity and specificity of the traditional cluster‐extent thresholding method in neuroimaging analyses, we here used the threshold‐free cluster enhancement (TFCE) statistical method. The TFCE model was introduced by Smith and Nichols in 2009 [23] and has since become a widely used method in neuroimaging studies. By enhancing the local maxima of the statistical map, this model enhances the sensitivity of the traditional cluster‐extent thresholding method, allowing for smaller clusters to be detected that would otherwise be missed by traditional methods. One advantage of this model is that it does not require a prespecified threshold, thereby making it a more data‐driven approach to thresholding [24]. The significant threshold was set to *P* < 0.05 corrected for family‐wise error for correlation analyses, and the voxel extent threshold was set to 50. Brain regions were identified using the Atlas of Neuromorphometrics implemented in SPM 12.

### 2.4 Statistical Analysis

Using Fisher's exact test, differences in the percentages of core clinical features between the Anxiety+ and Anxiety− groups were examined. Using the Spearman rank correlation coefficient, correlations between the NPI anxiety score, demographic characteristics, and clinical evaluation scores were examined. The significance level for all comparisons was set at *P* < 0.05. Statistical analysis was performed using IBM SPSS 28.0 software (IBM, Armonk, NY, USA).

## 3 Results

### 3.1 Participants

Of the 40 patients with DLB, 14 and 26 were categorized into the Anxiety+ and Anxiety− groups, respectively.

### 3.2 Results of Demographic Characteristics and Clinical Evaluations

Comparison of demographic characteristics and clinical evaluations between the Anxiety+ and Anxiety− groups showed no significant differences in each item (Table 1).

**TABLE 1:** Demographic characteristics and clinical evaluations.

	Anxiety+ *N* = 14	Anxiety− *N* = 26	*p* value
Age	76.1 ± 5.0	79.9 ± 6.1	0.055
Sex (male/female)	6/8	14/12	0.741
CDR (0.5/1)	7/7	17/9	0.500
MMSE total	21.4 ± 2.8	23.4 ± 3.3	0.130
Serial sevens test	2.4 ± 1.2	2.4 ± 1.7	0.989
Double pentagon (pass/fail)	12/2	20/6	0.689
ADAS total	13.3 ± 4.7	13.8 ± 5.2	0.944
Memory	8.3 ± 2.2	8.2 ± 3.1	0.790
Language	0.2 ± 0.6	0.7 ± 1.2	0.457
Act	3.1 ± 2.7	3.4 ± 2.3	0.644
Orientation	1.7 ± 1.3	1.5 ± 1.6	0.457

*Note:* Differences in the percentages of sex, CDR categories (0.5 or 1), and performance on the Double pentagon copying task (pass/fail) between the Anxiety+ and Anxiety− groups are examined using Fisher's exact test. Other items are examined using Mann–Whitney *U* test.

Abbreviations: CDR, clinical dementia rating; MMSE, Mini‐Mental State Examination; ADAS, Alzheimer's Disease Assessment Scale.

### 3.3 Relationship Between Anxiety and the 4 Core Clinical Features

The Anxiety+ group had significantly higher percentages of patients with fluctuating cognition than the Anxiety− group (*P* < 0.05; Table 2). No significant differences in the incidences of visual hallucinations, RBD, and parkinsonism were observed between the two groups.

**TABLE 2:** Comparison of the incidence of the 4 core clinical features between Anxiety+ and Anxiety− groups in DLB.

	Whole *N* = 40	Anxiety+ *N* = 14	Anxiety− *N* = 26	*p* value
Fluctuating cognition	55.0%	78.6%	42.3%	**0.046**
Visual hallucinations	65.0%	71.4%	61.5%	0.730
REM sleep behavior disorder	35.0%	42.9%	30.8%	0.501
Parkinsonism	47.5%	50.0%	46.2%	1.000

*Note:* Differences in percentages of the core clinical features between the Anxiety+ and Anxiety− groups are examined using Fisher's exact test. Statistically significant differences are shown in bold (*p* < 0.05).

Abbreviations: DLB, dementia with Lewy bodies; REM, rapid eye movement.

### 3.4 Relationship of Anxiety With Age and Cognitive Impairment

In the patients with DLB, the NPI anxiety score was significantly negatively correlated with age (*P* < 0.05; Table 3). However, it was not significantly correlated with any other evaluations.

**TABLE 3:** Relationship of anxiety with age and cognitive impairment in DLB.

	rs	*p* value
Age	−0.319	**0.045**
MMSE total	−0.298	0.061
Serial sevens test	−0.054	0.743
ADAS total	0.062	0.703
Memory	0.094	0.563
Language	−0.097	0.551
Act	−0.011	0.947
Orientation	0.119	0.463

*Note:* Correlations of the NPI anxiety score with age and cognitive impairment are examined using the Spearman rank correlation coefficient. Statistically significant correlations are shown in bold (*p* < 0.05).

Abbreviations: DLB, dementia with Lewy bodies; MMSE, Mini‐Mental State Examination; ADAS, Alzheimer's Disease Assessment Scale; NPI, Neuropsychiatric Inventory.

### 3.5 Correlations Between Anxiety and rCBF

**FIGURE 1:**
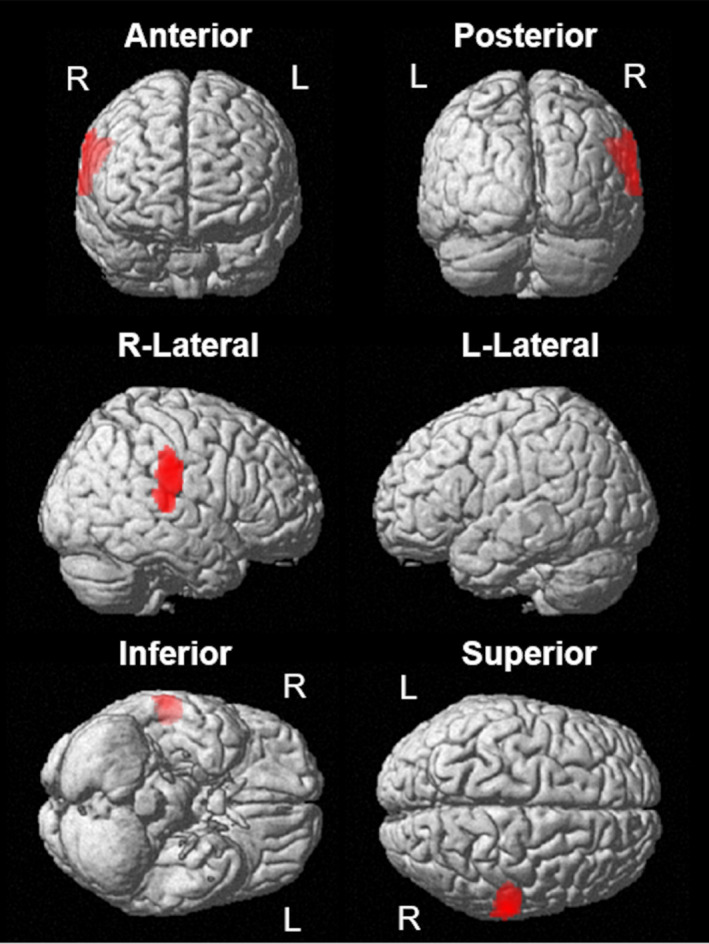
Correlation analysis between the NPI anxiety score and rCBF using SPM. Regions (right supramarginal gyrus) colored in red show that the NPI anxiety score is significantly negatively correlated with rCBF in DLB. The significant threshold is set to *p* < 0.05 (TFCE, FWE corrected) for the correlation analyses. DLB, dementia with Lewy bodies; FWE, family‐wise error; NPI, Neuropsychiatric Inventory; rCBF, regional cerebral blood flow; SPM, Statistical Parametric Mapping; TFCE, threshold‐free cluster enhancement.

In the SPM analysis using SPECT data, covariates included age, sex, CDR, SPECT model, and presence or absence of fluctuating cognition. The NPI anxiety score was significantly negatively correlated with rCBF in the right supramarginal gyrus of the patients with DLB (*P* < 0.05, FWE corrected; Figure 1). However, it was not significantly and positively correlated with rCBF in any of the brain regions of the patients with DLB.

## 4 Discussion

In this study, anxiety in patients with DLB was observed to be significantly negatively correlated with rCBF in the right supramarginal gyrus after adjustment for age, sex, CDR, SPECT model, and presence or absence of fluctuating cognition. This is the first study to report the neural basis of anxiety in patients with DLB using statistical imaging analysis. The right inferior parietal lobule (IPL), which includes the right supramarginal gyrus, plays a role as an inhibitory control mechanism in emotion regulation and is the brain region that decreases in function in individuals with anxiety disorders [25]. The IPL has a strong functional connectivity with the frontal lobes [26] and its impairment has been interpreted as a disturbance of specific regulatory mechanisms to anxious emotion in conjunction with the frontal lobes [27]. Furthermore, DLB is a disorder in which the function from the parietal to the occipital region declines compared with that of the frontal region [28]. Moreover, DLB reduces the rCBF at the back of the right parietal lobe [29]. The present findings that reduced function of the right supramarginal gyrus is significant for the appearance of anxiety in DLB are potentially influenced by the neurofunctional characteristics of the disease, with the parietal lobes being more likely impaired. It has been reported that by activating specific brain regions, repetitive transcranial magnetic stimulation (rTMS) can alleviate anxiety in generalized anxiety disorder and posttraumatic stress disorder [30], as well as BPSDs in dementia [31]. Thus, the activation of the right supramarginal gyrus by rTMS may alleviate anxiety in patients with DLB.

In this study, patients with DLB with anxiety demonstrated significantly higher rates of fluctuating cognition than those without anxiety, suggesting that fluctuating cognition is closely associated with anxiety in DLB. This finding suggests that fluctuating cognition can trigger anxiety in these patients. Notably, fluctuating cognition in DLB is associated with delirium, with cholinergic dysfunction and disturbances in arousal and attention systems thought to cause both conditions [32]. Furthermore, previous studies have indicated that anxiety in DLB is frequently observed during episodes of delirium [15] and that patients who experience delirium in the acute phase of stroke are more likely to develop anxiety in later stages [33]. These findings support the interpretation that fluctuating cognition plays a causal role in the occurrence of anxiety in DLB. However, our findings also suggest that anxiety can trigger or exacerbate fluctuating cognition in patients with DLB, which has been linked to attentional impairments [32]. As described in the diagnostic criteria for DLB, fluctuating cognition is characterized by marked fluctuations in attention [3]. It is well established that anxiety disrupts the efficient functioning of goal‐directed attentional systems [34]. Furthermore, in cognitively healthy older adults, higher anxiety levels have been linked to lower cognitive performance [35]. Overall, these results support the alternative interpretation that anxiety may contribute to the emergence or worsening of fluctuating cognition in DLB. The potential bidirectional relationship identified in this study points to possible bidirectional therapeutic strategies. Fluctuating cognition in DLB has been demonstrated to improve with donepezil treatment [36]. When a causal relationship exists wherein fluctuating cognition induces anxiety, cholinesterase inhibitors may indirectly help alleviate anxiety by stabilizing the fluctuating cognition. Conversely, when anxiety contributes to the occurrence of fluctuating cognition, nonpharmacological interventions, including supportive care and engagement aimed at reducing anxiety, may also reduce fluctuating cognition.

In this study, anxiety was significantly negatively correlated with age in the patients with DLB. Anxiety in patients with dementia is psychosocially regarded as an early compensatory emotional response to difficulties in adjusting to daily life [37]; In particular, anxiety in younger dementia patients is associated with the importance of social and familial roles [38]. Moreover, memory impairment in younger patients with DLB is often mild [39]. Therefore, younger patients with DLB may experience higher anxiety levels as they remember more failures in their daily lives.

In this study, no significant difference in the percentage of patients exhibiting visual hallucinations was noted between the Anxiety+ and Anxiety− groups. The only study suggesting an association between anxiety and visual hallucinations in DLB reported that experimentally inducing an anxious mood triggered pareidolic illusions in patients with DLB [14]. However, that study did not directly analyze the association between anxiety and visual hallucinations. The pareidolia test measures illusion‐like phenomena under experimental conditions and may not accurately reflect actual visual hallucinations, as even patients with DLB without visual hallucinations can exhibit pareidolic responses [40]. Moreover, visual hallucinations in DLB are influenced by anxiety and other factors, including sleep disturbances [41] and visuospatial cognitive impairments [42]. Therefore, the nature of visual hallucinations may partly explain why a consistent association with anxiety has not been observed. In addition, in this study, identifying visual hallucinations relied on family observations; therefore, the likelihood of overlooking some visual hallucinations could not be ruled out.

In this study, no significant differences were demonstrated in the performance on the MMSE double pentagon copying task between the Anxiety+ and Anxiety− groups. As previously mentioned, anxiety was significantly associated with the right parietal lobe. The right parietal lobe plays a role in visuoconstructional ability [43]. The lack of a significant association observed in this study may be attributed to two reasons. One explanation is that the sensitivity of the MMSE double pentagon copying task may be limited, potentially failing to detect subtle deficits in visuoconstructional ability. Furthermore, as this study encompassed patients with CDR scores of 0.5 and 1, several patients demonstrated good performance. Therefore, visuoconstructional ability, as assessed using the MMSE double pentagon copying task, may be relatively preserved in the early stages of DLB, regardless of the presence or absence of anxiety.

This study had some limitations. First, this study was based on data from two institutions, and the sample size was small. Second, fluctuating cognition was only evaluated on the basis of its presence or absence. Third, in the SPM analysis, if there were symptoms among the 4 core features associated with anxiety, the presence or absence of those symptoms would also be included in the covariates. This was done to cope with the small sample size, which forced us to reduce the number of covariates. Fourth, the causal relationship between anxiety and fluctuating cognition could not be determined within the framework of this study. Fifth, although the diagnosis and clinical assessments of DLB were evaluated in detail by a skilled dementia specialist, this study is classified as a retrospective study. Lastly, the diagnosis of DLB was not pathologically confirmed. To confirm and expand upon these findings, future studies with larger and more demographically and clinically diverse DLB cohorts are warranted.

## 5 Conclusion

Anxiety in DLB is associated with reduced rCBF in the right supramarginal gyrus and is also related to fluctuating cognition. The former finding that impaired function of the right supramarginal gyrus may be interpreted as a disturbance of inhibitory regulatory mechanisms for anxiety. However, the causality underlying the latter finding could not be determined in this study. Further research is warranted to elucidate this causal relationship because it may contribute to the development of effective therapeutic strategies.

## Author Contributions

Naohiro Kimura and Hiroaki Kazui designed the study. All authors supervised the data collection. Naohiro Kimura, Yoshihiro Chadani, Hideki Kanemoto, Ryuichi Takahashi, Tetsuo Kashibayashi, and Kenji Tagai assisted in the analysis of imaging data. Naohiro Kimura was responsible for the statistical design and analysis and wrote the first manuscript draft. All authors were involved in the interpretation and presentation of the data, reviewed and revised the initial draft and subsequent versions of the manuscript. Hiroaki Kazui approved the final version manuscript.

## Consent

Since this study was a retrospective study, an opt‐out method was adopted.

## Conflicts of Interest

The authors declare no conflicts of interest.

## Supporting information


Table S1

## Data Availability

The data supporting the findings of this study are available on request from the corresponding author.
