# Supplementary material for: Neural Basis of Anxiety in Dementia With Lewy Bodies
Source: Int J Geriatr Psychiatry. 2025 Sep 3;40(9):e70150. doi: 10.1002/gps.70150 (PMC12406763; doi:10.1002/gps.70150)
Supplement: Supplementary file 1 — Table S1 [file GPS-40-e70150-s001.pptx]

## Slide 1
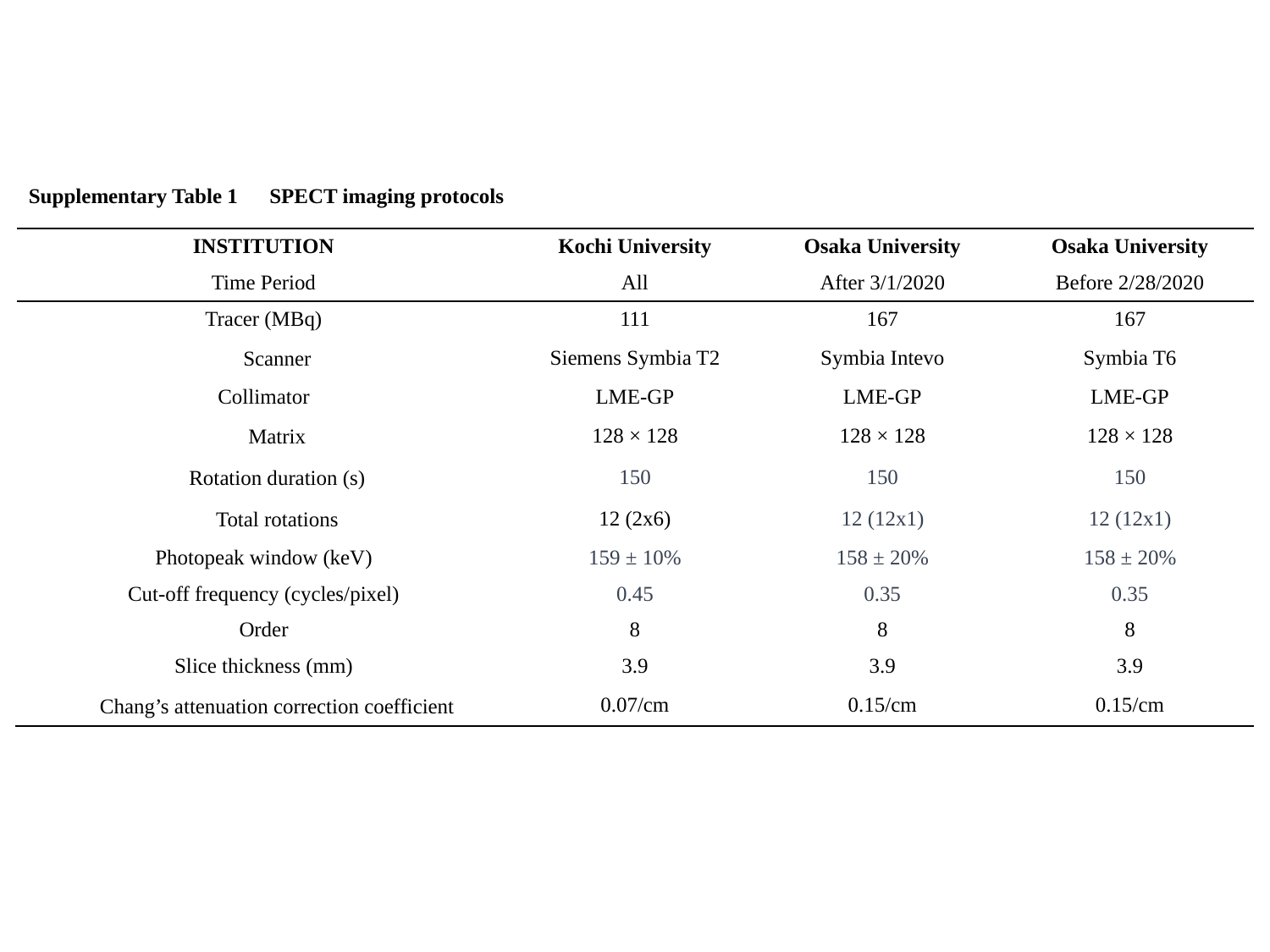

Supplementary Table 1　SPECT imaging protocols
| INSTITUTION | Kochi University | Osaka University | Osaka University |
| --- | --- | --- | --- |
| Time Period | All | After 3/1/2020 | Before 2/28/2020 |
| Tracer (MBq) | 111 | 167 | 167 |
| Scanner | Siemens Symbia T2 | Symbia Intevo | Symbia T6 |
| Collimator | LME-GP | LME-GP | LME-GP |
| Matrix | 128 × 128 | 128 × 128 | 128 × 128 |
| Rotation duration (s) | 150 | 150 | 150 |
| Total rotations | 12 (2x6) | 12 (12x1) | 12 (12x1) |
| Photopeak window (keV) | 159 ± 10% | 158 ± 20% | 158 ± 20% |
| Cut-off frequency (cycles/pixel) | 0.45 | 0.35 | 0.35 |
| Order | 8 | 8 | 8 |
| Slice thickness (mm) | 3.9 | 3.9 | 3.9 |
| Chang’s attenuation correction coefficient | 0.07/cm | 0.15/cm | 0.15/cm |
